# Long non-coding RNAs have age-dependent diurnal expression that coincides with age-related changes in genome-wide facultative heterochromatin

**DOI:** 10.1186/s12864-018-5170-3

**Published:** 2018-10-29

**Authors:** Jinhee Park, William J. Belden

**Affiliations:** Department of Animal Sciences, Rutgers, The State University of New Jersey, New Brunswick, NJ 08901 USA

**Keywords:** Circadian rhythm, Aging, Long non-coding RNA, Diurnal, Heterochromatin, Histone H3 lysine 9 methylation

## Abstract

**Background:**

Disrupted diurnal rhythms cause accelerated aging and an increased incidence in age-related disease and morbidity. The circadian clock governs cell physiology and metabolism by controlling transcription and chromatin. The goal of this study is to further understand the mechanism of age-related changes to circadian chromatin with a focus on facultative heterochromatin and diurnal non-coding RNAs.

**Results:**

We performed a combined RNA-seq and ChIP-seq at two diurnal time-points for three different age groups to examine the connection between age-related changes to circadian transcription and heterochromatin in neuronal tissue. Our analysis focused on uncovering the relationships between long non-coding RNA (lncRNA) and age-related changes to histone H3 lysine 9 tri-methylation (H3K9me3), in part because the Period (Per) complex can direct facultative heterochromatin and models of aging suggest age-related changes to heterochromatin and DNA methylation. Our results reveal that lncRNAs and circadian output change dramatically with age, but the core clock genes remain rhythmic. Age-related changes in *clock-controlled gene (ccg)* expression indicate there are age-dependent circadian output that change from anabolic to catabolic processes during aging. In addition, there are diurnal and age-related changes in H3K9me3 that coincide with changes in transcription.

**Conclusions:**

The data suggest a model where some age-related changes in diurnal expression are partially attributed to age-related alterations to rhythmic facultative heterochromatin. The changes in heterochromatin are potentially mediated by changes in diurnal lncRNA creating an interlocked circadian-chromatin regulatory network that undergoes age-dependent metamorphosis.

**Electronic supplementary material:**

The online version of this article (10.1186/s12864-018-5170-3) contains supplementary material, which is available to authorized users.

## Background

Early circadian research showed non-circadian day lengths negatively impact longevity [1], even though the free-running rhythm only changes slightly with age [2]. More recent reports indicate circadian disruption contributes to increased morbidity [3] and diseases including; metabolic disorders [4–6], depressive disorders [7], cardiovascular disease [8], cancer [9], and advanced aging [10]. In addition, age-related changes to the circadian clock result in a dampened endocrine and neuroendocrine hormone rhythm [11] concurrent with a decrease in *Clock* and *Bmal1* expression, while the rhythm in *Per* genes is largely unaffected [12]. There are also extensive age-related changes to *clock-controlled genes* (*ccg*) in the liver that correlate with age-related changes to metabolism which occurs with a change in acetylation [13, 14]. As a result of these reports and others, there is a growing appreciation of the connection among circadian disruption, advanced-aging and an increased incidence of age-related maladies, yet the mechanism underlying these remains cryptic.

The core circadian rhythm is maintained by a transcriptional negative feedback loop where the dimeric transcriptional activators CLOCK and BMAL1 [15–18] drive expression of the *Period (Per1, Per2, Per3)* [19–21] and *Cryptochrome* (*Cry1, Cry2*) genes [22, 23]. Once expressed, the PERs associate with the CRYs and form a multisubunit complex (approximately 2 MDa) that contains repressive chromatin modifiers including a histone H3 lysine 9 methyltransferase (KMT1/Suv39h), RNA binding proteins, and the nucleosome remodeling and deacetylase (NuRD) corepressor [24–27]. In a second feedback loop, RORα and REV-ERBα have a positive and negative role, respectively, in regulating rhythms in *Bmal1* expression [28, 29]. The clock regulates, either directly or indirectly, a large percentage of protein-coding genes [30–36] and controls genome-wide chromatin states that oscillate between permissive and non-permissive states [31, 32, 37]. Facultative heterochromatin at the central clock gene(s) in Neurospora*,* Drosophila and mammals is observed during the repressive phase and is characterized by histone H3 lysine 9 di- and tri-methylation (H3K9me2 & H3K9me3) and HP1 binding [24, 38–42]. The extent of circadian-regulated facultative heterochromatin (CRFH) throughout the genome is unknown, but it does occur at *D-element binding protein* (*Dbp*) suggesting, at the very least, it is present at a *ccg* [41].

Long non-coding RNAs (lncRNAs) and natural antisense transcripts (NATs) are known to aid in establishing repressive chromatin and can function in both *cis* and *trans* [43, 44]. As such, they are a potential mechanism by which the circadian clock could establish permissive or non-permissive chromatin states to regulate gene expression. Support for this idea comes from numerous reports documenting a wide-array of rhythmic ncRNA [32] including a NAT that originates from *Per2* [31, 32]. Based on studies on the Neurospora *frequency* NAT, it is possible that *Per2AS* may be necessary to help establish heterochromatin and assist in feedback repression [39].

Current theories on aging suggest that changes in heterochromatin may be responsible for age-related changes in gene expression and alterations in H3K9me3 may function as a driver of aging [45, 46]. For example, in models of Werner syndrome, a premature aging disorder in humans, there are genome-wide changes to H3K9me3. The changes in H3K9me3 in Werner syndrome are likely due to WRN-guided heterochromatin because WRN associates with KMT1/SUV39H and HP1 [46, 47]. The connections among the circadian clock, heterochromatin, lncRNA and aging led us to examine the role of lncRNA in age-related changes to the clock-chromatin circuit and ascertain whether this may contribute to age-related diseases. To accomplish this, we performed a comprehensive RNA-sequencing (RNA-seq) and H3K9me3 ChIP-seq on zebrafish brain tissue from animals that were 4, 12, and 20 months (M) old at zeitgeber time (ZT) 4 and ZT16. We observed age-related changes to diurnal H3K9me3 and RNA expression, with a subset of diurnal genes showing changes in H3K9me3. The results suggest that age-related changes to circadian regulated transcription occur in part due to changes in heterochromatin.

## Results

### Identification of long non-coding RNAs

There are age-related changes to circadian transcription that coincide with changes in physiology, but the connection to changes in circadian noncoding RNA and chromatin are lacking. To further define age-related changes to circadian transcription, including changes in lncRNA expression, we performed a multi-dimensional RNA-sequencing (RNA-seq) experiment with a focus on lncRNA transcript discovery. We isolated total RNA from zebrafish brain tissue at two diurnal time points (ZT4 and ZT16) for 3 different ages (4 M, 12 M, 20 M). We processed the ribo-depleted, stranded RNA-seq data using Tophat2/Cufflinks (T/C) [48] and/or HISAT2/StringTie (H/S) [49] with the zebrafish genome GRCz10. In our analyses, we also included stranded RNA-seq data from developing zebrafish embryos [50] to enhance lncRNA identification. In order to detect the known *per2AS* transcript with H/S pipeline, we had to use an FPKM cut-off of 0.1 in StringTie. Stipulating *per2AS* identification as criteria in StringTie generated an excessive number of single-exon transcripts (> 150,000), so we proceeded with the analysis using T/C results (Additional files 1 and 2). The Cufflinks transcripts were further subdivided by class code and processed with bedtools intersect to identify potential overlapping lncRNA. This analysis revealed there were potentially 14,773 overlapping transcripts (OT). Next, we processed the OT with PLEK (**p**redictor of **l**ong non-coding RNAs and m**e**ssenger RNAs based on an improved **k**-mer scheme) to predict potential NATs [51] with the notion these may play a role in chromatin regulation. Overall, this analysis revealed 5299 lncRNAs overlapped with another gene, either protein-coding gene or overlapping lncRNAs. The inclusion of RNA-seq data from developing zebrafish embryos allowed us to perform a comprehensive analysis of lncRNA from early development through aged adult animals. To further parse the data, we took the 146,910 transcripts from Cufflinks and found 87,327 transcripts that are not contained in zebrafish GRCz10 genome assembly. These 87,327 transcripts were processed with PLEK to find lncRNA [51] yielding 50,524 potential lncRNAs. Next, we filtered the 50,524 potential lncRNAs based on expression stipulating that a transcript must change in at least one sample set to separate regulated transcription versus nonspecific transcriptional noise. In total, 17,702 potential lncRNAs displayed differential expression from embryogenesis to aged adult. Further clustering of 17,702 transcripts into 8 groups based on expression revealed that expression of some lncRNA are restricted to specific times during embryo development, while others are restricted to adult fish (Additional file 3). In general, we found specific lncRNAs are largely confined to brain tissue and were either not present during development or were low abundance. One group (Group 6, Additional file 3 g**)** displayed a noticeable diurnal oscillation at 12 M where transcripts were highly expressed at ZT4, but largely absent at ZT16. These same lncRNAs appeared constant regardless of the time-of-day at other ages.

### Age-related changes to diurnal gene expression

**Fig. 1:**
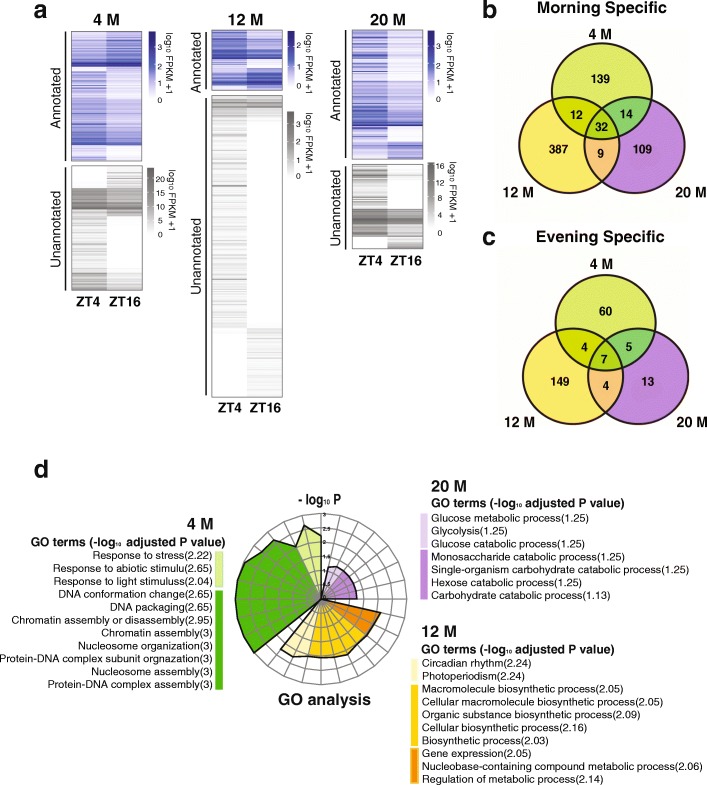
Age-related changes in diurnal transcription of zebrafish brain tissue. **a** Heatmaps illustrate diurnal regulated genes from zebrafish brain tissue at 4 M, 12 M and 20 M (FDR ≤0.05). Transcripts were sorted into annotated (blue color scale) and unannotated genes (gray color scale). The list of genes and locus information is contained in Additional file 4 :Tables S1a-c. The overlap of age-related changes in diurnal gene expression was determined for (**b**) morning-specific genes (elevated at ZT4) and (**c**) evening-specific genes (elevated at ZT16). **d** Radial graph depicting the GO analysis from age-specific diurnal genes (*P* ≤ 0.01). Functionally related GO terms are clustered based on age (4 M; green, 12 M; yellow and 20 M; purple)

Next, we proceeded to examine diurnal expression across all three age groups. Transcripts were split into protein-coding genes and transcripts not currently annotated in the GRCz10 reference assembly (unannotated transcripts). Among the 87,327 unannotated transcripts, PLEK predicted roughly 58% were lncRNA (Additional file 3). Analysis of diurnal expression comparing ZT4 to ZT16 (q ≤ 0.05) revealed 273, 604, and 193 diurnal transcripts in 4, 12, and 20 M age groups, respectively (Fig. 1a). Unannotated transcripts represented 48.7% and 39.4% in 4 and 20 M groups, while they represented 83.4% of diurnally expressed transcripts in the 12 M group. The annotated diurnal genes for each age group can be found in Additional files 4-6. At each age, a portion of the diurnal transcripts overlapped with another transcript on the opposite DNA strand. In total, 9.5% (26 of 273), 6.6% (40 of 604), and 12.4% (24 of 193) of diurnal transcripts at 4, 12, and 20 M contained an overlapping transcript.

**Fig. 2:**
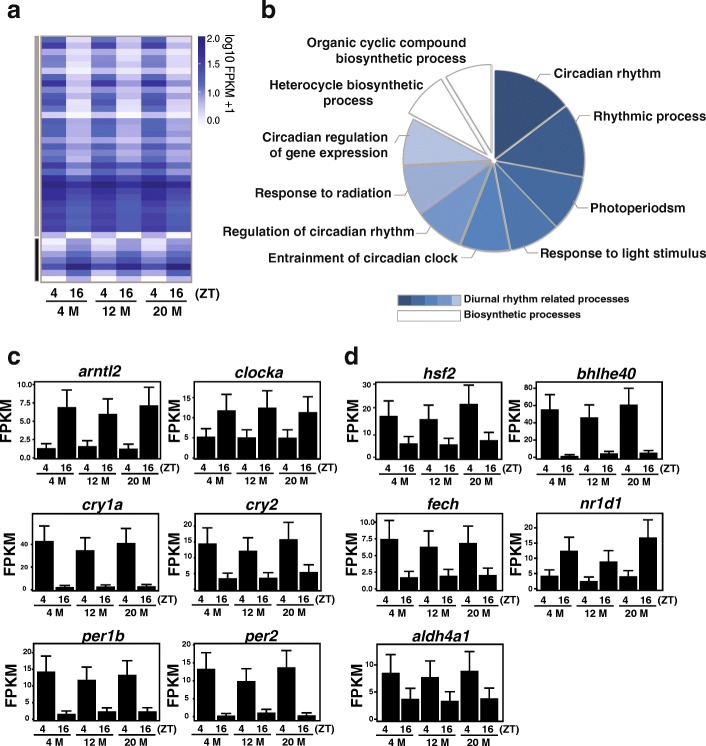
The core clock remains rhythmic regardless of age. **a** Expression of the 39 genes that maintained oscillations across all age groups. **b** Functional categorization of the GO terms for the 39 genes whose diurnal expression did not change with age. Genes related to diurnal rhythm-related function (shaded blue) or other biosynthetic processes (shaded white). **c** Relative expression at both times across all age groups for a subset of genes that did not change with age (FDR < 0.05)

To examine age-related changes in diurnal gene expression, diurnally expressed genes from the three different ages were split into two groups. Those that peaked at ZT4 were classified as morning-specific and those that peaked at ZT16 were classified as evening-specific. Comparison across ages revealed the vast majority of diurnal genes change with age and only a small subset (32 morning-specific and 7 evening-specific) of genes maintained diurnal expression regardless of age (Fig. 1b,c). The morning-specific genes included *per2* and *cry1a*, while evening-specific genes included *bmal1/arntl1a* and *clock* (Fig. 2c). We further examined the age-related changes to diurnal gene expression by clustering transcripts elevated at ZT4 relative to ZT16 for each age (Additional file 7). In general, morning and evening specific genes changed expression as the animals aged.

To further define the physiological consequence of age-related changes in diurnal gene expression, we calculated gene ontology (GO) enrichment as a function of age using WEB-based GEne SeT AnaLysis (WebGestalt) Toolkit [52, 53] (Fig. 1d). This analysis showed diurnal genes differed in their ontology as a function of age. For example, in 4 M-old fish diurnally expressed genes were mainly associated with nucleosome/chromatin assembly, protein-DNA complex assembly and response to stimuli such as light, abiotic factors, and stress. In 12 M fish, the top enriched GO terms included circadian rhythm along with biosynthetic and metabolic processes. In 20 M fish, the most significant GO terms included glucose metabolism and carbohydrate or monosaccharide catabolism. The results indicate that age-related changes in diurnal genes are chronologically clustered into different biological processes that include cellular growth and anabolic processes in young animals, changing to genes involved in cellular maintenance in adults, and switching to catabolism in older animals.

Next, we performed disease enrichment on diurnal-regulated genes at each age to compare age-related changes with known disease markers. Disease Ontology (DO) analysis was conducted using DOSE [54] and revealed a correlation between gene expression and age-related diseases (Additional file 8). In addition to mood and bipolar disorder that appeared to manifest in older animals, genes implicated in diseases originating in other tissues were also present, suggesting that separate alterations in clock output in peripheral tissue may be a causative event in disease manifestation. Furthermore, we used the Age-Related Disease (ARD) database [55] and found an association between gene expression changes and age-related cognitive decline. For example, we found that *c3a.1* and *anxa3b*, genes implicated in neurodegenerative diseases such as Alzheimer’s disease and Parkinson’s [56], lost their diurnal rhythm at 20 M (Additional file 8b).

### Circadian clock remains constant regardless of age

We next focused on the 39 genes (32 morning-specific and 7 evening-specific) that maintained diurnal expression regardless of age (Fig. 2a). GO analysis revealed the vast majority of genes are involved in circadian clock regulation (Fig. 2b). This subset of genes includes *arntl2*, *clocka*, *cry1a, cry2, per1b* and *per2* as well as *hsf2*, *bhlhe40*, *fech*, *nr1d1* and *aldh4a1,* which all show distinctive diurnal expression (Fig. 2c). Consistent with other reports [13, 14], these data indicate that core clock genes are largely unaffected by age, suggesting there is an undefined age-related network that alters circadian output beyond the normal plasticity found in circadian oscillators.

### Age-specific diurnal genes with overlapping transcripts

**Fig. 3:**
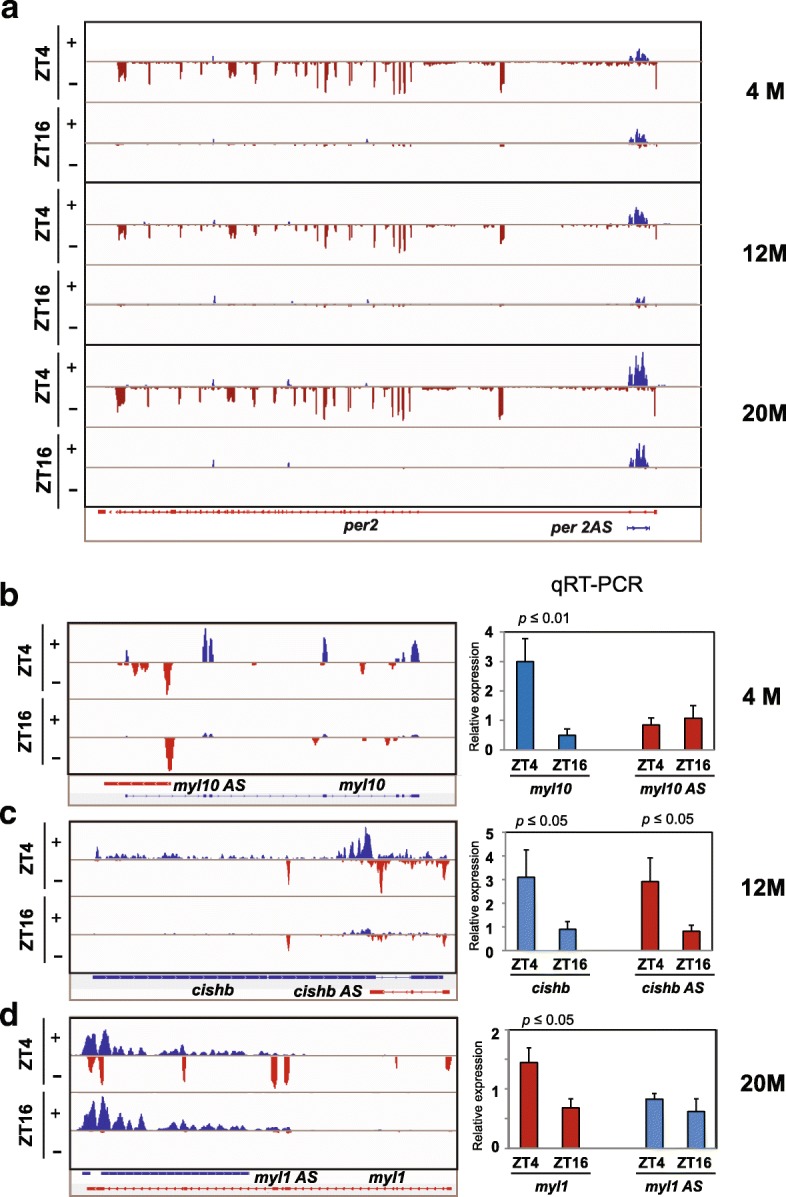
Representative examples of overlapping transcripts. IGV traces of the (**a**) *per2* locus showing relative expression of transcripts that originate from the plus strand (blue) and the minus strand (red) across all three age-groups. Representative IGV traces and validation of sense and antisense transcripts by quantitative RT-PCR of diurnal transcripts with corresponding antisense transcripts for each representative age-group (**b**) *myl10* at 4 M, (**c**) *cishb* at 12 M and (**d**) *myl1* at 20 M. Error bars present SEM and the *p*-values are shown on the graphs

A fraction of diurnally expressed genes had a corresponding overlapping transcript so we examined how the expression of these pairs changed with age with the idea that they may engage in transcriptional interference of represent a chromatin-regulating lncRNA and it’s cognate *cis* target. This concept is rooted in the observation that lncRNAs are known regulators of chromatin and can influence gene expression in both *cis* and *trans* [44]. In support of this notion, we found that 9.5, 6.6 and 12.4% of diurnal genes for the 3 age groups had an OT. Expression of representative diurnal genes with corresponding OT is in shown in Fig. 3a-d. As previously noted *per2*, which maintained a diurnal rhythm regardless of age, has a corresponding antisense transcript (*per2AS*), although in the RNA-seq *per2AS* lacks a readily apparent rhythm for the 2 diurnal time points tested here (Fig. 3a). However, when we examine a 24-h time course using strand-specific RT-PCR an antiphasic rhythm is apparent (data not shown). Diurnal expression of other genes with OT was restricted to specific ages. For example, *myl10* has diurnal expression at 4 M but not 12 and 20 M. Like *per2AS*, *myl10AS* does not have a detectible rhythm at any age (Fig. 3b, Additional file 9a). In contrast, *cishb* and *cishbAS* both have diurnal expression with a similar phase (both transcripts are elevated at ZT4 and reduced at Z16) (Fig. 3c, Additional file 9b). Finally, *myl1* exhibited diurnal expression at 20 M, but not 4 M and 12 M and its OT, *myl1AS* did not show a detectible rhythm at any age examined (Fig. 3d, Additional file 9 c). RT-PCR results for these OT are consistent with the RNA-seq data, confirming OT are present at a subset of diurnal genes but do not necessarily display antiphasic rhythms; a finding consistent with results from liver [32]. However, our data are limited to 2 diurnal time-points, so we cannot rule out the possibility that these OTs have an oscillation with a different phase. In addition, the antisense transcripts were all expressed at much lower levels so detecting differences was inherently more difficult.

### Diurnal and age-related changes to facultative heterochromatin

**Fig. 4:**
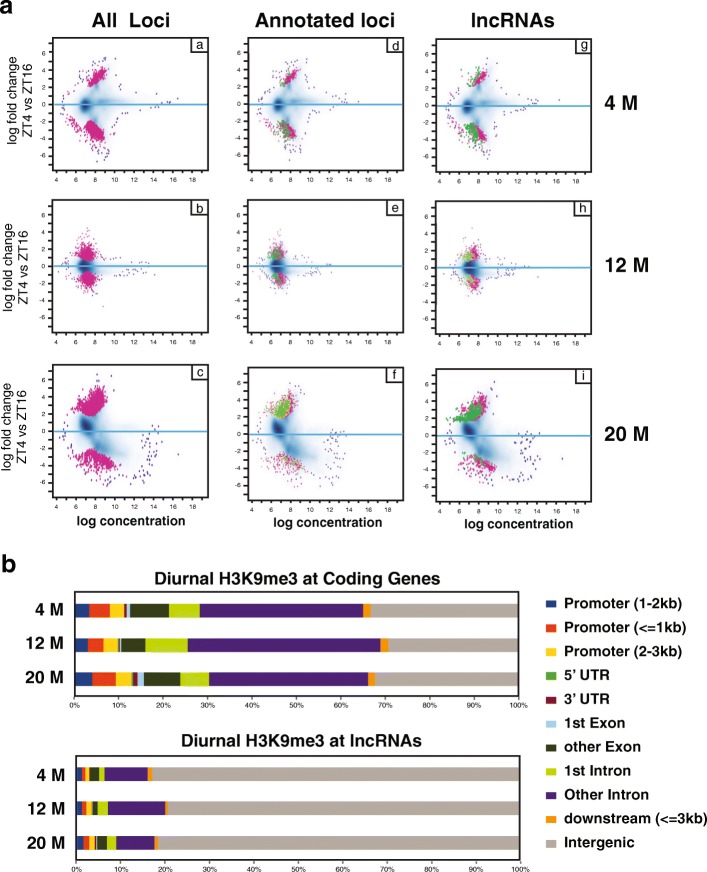
Diurnal rhythm in genome-wide H3K9me3 for each age group. **a** Diurnal changes in H3K9me3 levels between ZT4 and ZT16 for all 3 age groups. Loci with significant diurnal changes in H3K9me3 are shown in red. Positive values indicate H3K9me3 peaking at ZT4 and negative values peak at ZT16. The plots are further subdivided into annotated loci or predicted lncRNAs. The green spots indicate loci with OT. **b** Relative genome feature location of diurnal H3K9me3 for coding genes and lncRNAs

Given our observation of many diurnal lncRNAs and their potential role as regulators of chromatin, we proceeded to examine if there were age-related changes in facultative heterochromatin, specifically H3K9me3, which might account for the age-related changes in gene expression. We performed H3K9me3 chromatin immunoprecipitation (ChIP) from crosslinked zebrafish brain tissue at ZT4 and ZT16 at 4, 12 and 20 months of age. Chromatin enriched with H3K9me3 was identified using MACS2 (q ≤ 0.01) [57] and differential diurnal distribution within and between ages defined by DiffBind [58]. Diurnal heterochromatin between ZT4 and ZT16 was observed at all ages (*p* ≤ 0.05) and H3K9me3 levels changed throughout the genome concurrent with change in age. For example there were 3424 rhythmic H3K9me3 peaks at 4 M, 3417 at 12 M and 9717 at 20 M (Fig. 4a a-c). To understand the distribution of H3K9me3 between coding and non-coding loci and the extent to which H3K9me3 oscillated at OT, the H3K9me3 peaks were broken down into those occurring in or near protein-coding genes (Fig. 4a d-f) or at loci containing predicted lncRNA (Fig. 4a g-i). The peaks were further classified into ones that occurred near OTs and these are highlighted in green. At 4 M, 14.2% of the diurnal H3K9me3 peaks occurred at annotated genes with an OT, while 8.3% occurred at a lncRNA with an OT; at 12 M 22% occurred at annotated genes with an OT and 13.6% occurred at a lncRNA with an OT; and at 20 M, 11.2% of peaks occurred at annotated genes with an OT and 22.2% occurred at a lncRNA with an OT. We next examined the differences in genome location of H3K9me3 that occurred in protein-coding genes relative to regions containing lncRNA (Fig. 4b). At protein-coding genes, H3K9me3 tended to cluster in promoter regions 1–3 kb upstream of transcription start site, in introns and intergenic regions, whereas at lncRNAs, H3K9me3 mostly occurs in intergenic regions. This phenomena was likely observed because lncRNAs lack a 5′ or 3’ UTR and tend to be smaller with less splicing than their protein-coding counterparts. Collectively, the data indicate there are genome-wide diurnal oscillations in H3K9me3 that change in an age-dependent manner.

**Fig. 5:**
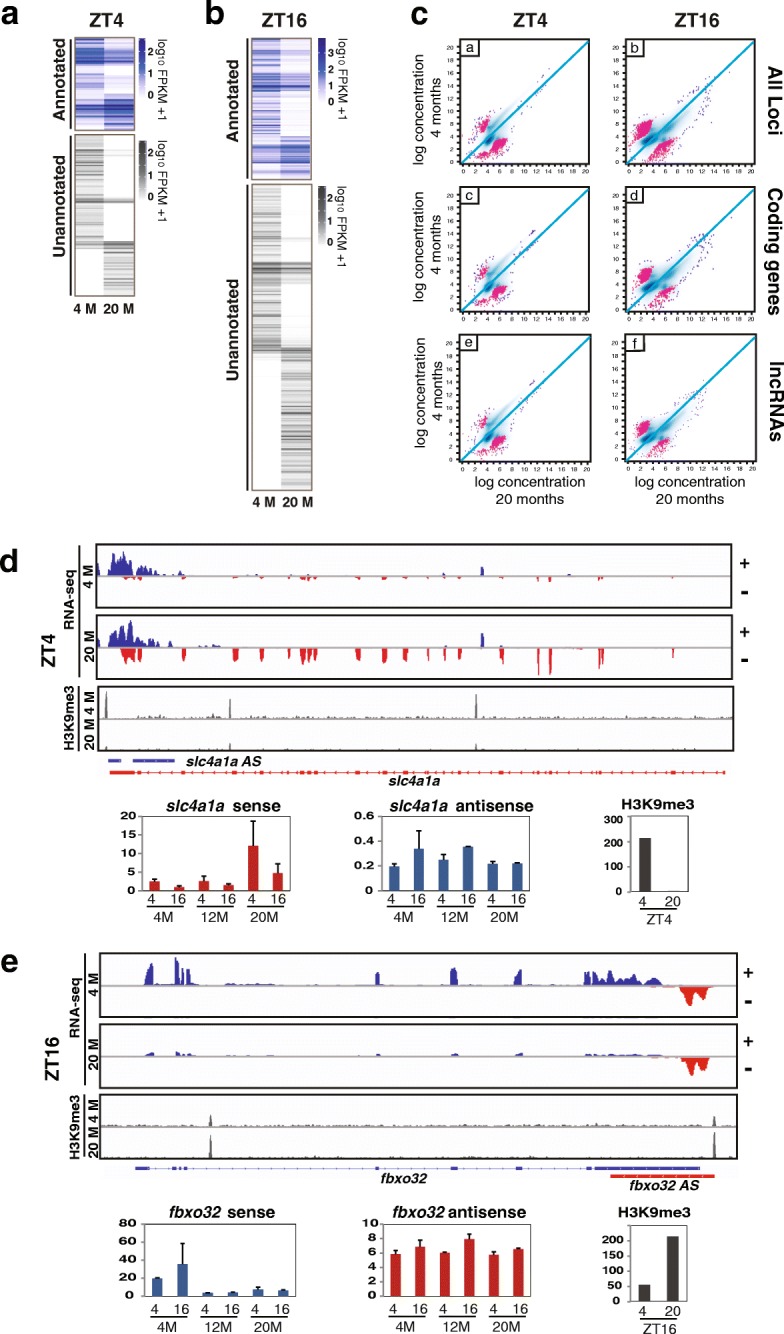
Age-related changes in transcript abundance coincide with changes in heterochromatin. **a** Genes that display age-related changes in expression between 4 M and 20 M at ZT4 (FDR ≤0.02). The genes are further subdivided into 50 annotated (blue scale) and 136 unannotated genes (Grayscale). **b** Same as in (**a**) except these loci have age-related changes at ZT16. At ZT16 there were 178 annotated genes (top) and 296 novel genes (bottom) that changed between 4 M and 20 M. Genes represented in the heatmaps are contained in Additional file 5: Tables S2a and b. **c** Scatter plots showing age-related changes in H3K9me3 between 4 M and 20 M at ZT4 and ZT16 for all loci described in (**a**) and (**b**). Loci with significant diurnal changes in H3K9me3 are shown in red. The data are further subdivided into protein-coding genes and lncRNAs. Representative examples of age-related changes in expression and H3K9me3 at genes with OT are visualized with IGV. **d** At ZT4, *slc4a4* has an age-related increase in expression and an age-related decrease in H3K9me3. **e** At ZT16 there is an age-related loss in *fbxo32* and a corresponding increase in H3K9me3

To further define age-related changes in facultative heterochromatin and examine the impact on age-related changes in gene expression, we began by selecting genes that displayed differential expression between 4 M and 20 M at the same time point (q ≤ 0.05). We found 266 transcripts had an age-related change at ZT4 and 462 at ZT16 (Fig. 5a, b). At ZT4, 76 annotated and 190 unannotated transcripts changed expression between 4 and 20 M and at ZT16, 167 annotated and 295 unannotated transcripts displayed age-related changes (Additional files 10 and 11). Next, we sorted the genes with age-related changes into four groups: Group 1 was morning-specific genes that decrease with age, Group 2 was morning-specific genes that increase with age, Group 3 was evening-specific genes that decrease with age and Group 4 was evening-specific genes that increase with age. Consistent with previous findings [14], GO analysis revealed age-related changes to metabolic function were prevalent (Table 1). Other notable findings in our analysis indicate that cardiovascular fitness and regenerative potential decline with age (Group 1) whereas responses to hypoxia and low oxygen are endemic to aged animals (Group 4).

**Table 1:** The most enriched GO clusters are selected from 4 groups of genes and ranked by their -log10 p-value (*p* ≤ 0.01)

GO annotation	adj *P* value
Morning-Specific	
Group 1: Decrease with Age	
heart contraction	3.70E-03
heart process	3.70E-03
fin regeneration	4.70E-03
circulatory system process	5.50E-03
blood circulation	5.50E-03
tissue regeneration	7.80E-03
fin morphogenesis	1.48E-02
purine-containing compound biosynthetic process	1.88E-02
response to wounding	1.93E-02
appendage development	1.93E-02
Group 2: Increase with Age	
short-chain fatty acid metabolic process	6.24E-05
regulation of biological quality	2.00E-04
homeostatic process	6.00E-04
fatty acid metabolic process	1.40E-03
ion homeostasis	1.70E-03
chemical homeostasis	2.20E-03
monocarboxylic acid metabolic process	3.10E-03
cellular lipid metabolic process	1.14E-02
carboxylic acid metabolic process	2.08E-02
oxoacid metabolic process	2.22E-02
Evening-Specific	
Group 3: Decrease with Age	
glycolysis	1.92E-06
monosaccharide metabolic process	3.32E-06
hexose metabolic process	3.32E-06
glucose catabolic process	4.92E-06
monosaccharide catabolic process	5.66E-06
single-organism carbohydrate catabolic process	5.66E-06
hexose catabolic process	5.66E-06
carbohydrate catabolic process	6.48E-06
glucose metabolic process	1.52E-05
generation of precursor metabolites and energy	2.00E-04
Group 4: Increase with Age	
endothelial cell migration	2.00E-04
immune system process	1.00E-03
response to decreased oxygen levels	1.10E-03
response to hypoxia	1.10E-03
response to oxygen levels	1.10E-03
sprouting angiogenesis	1.50E-03
erythrocyte homeostasis	2.70E-03
erythrocyte differentiation	2.70E-03
embryonic hemopoiesis	2.90E-03
homeostasis of number of cells	3.10E-03

We next examined age-related changes to H3K9me3. Analysis of H3K9me3 between 4 and 20 M suggested H3K9me3 levels change for large portions of the genome as organisms age (Fig. 5c). This analysis shows an overall decrease in significant H3K9me3 peaks in 4 M versus 20 M at ZT4 and the opposite at ZT16. Changes occurred at protein-coding genes and lncRNA and were particularly pronounced for lncRNAs, some of which contained sense-antisense pairs. When compared with gene expression, H3K9me3 enrichment generally showed the expected and opposite pattern. For example, H3K9me3 was elevated near *slc4a1a* at 4 M relative to 20 M and *slc4a1a* showed a corresponding decrease in expression at 20 M compared to 4 M (Fig. 5d, Additional file 12a-c). In this case, loss of H3K9me3 at ZT4 near *slc4a1a* with increasing age resulted in a gain of morning-specific diurnal expression that was exclusive to 20 M brain. Conversely, we also observed the opposite and found an age-related loss in diurnal expression and a corresponding increase in heterochromatin. This phenomenon is represented by *fbxo32* (Fig. 5e), which displayed a diurnal oscillation at 4 M in the RNA-seq that was lost at 12 and 20 M. In this instance H3K9me3 levels increased with age at ZT16, which corresponded with the loss of expression. Despite the age-related changes in diurnal expression of the protein-coding genes and heterochromatin, the corresponding overlapping lncRNA did not always show an overt rhythm or expression differences among ages, indicating there is far more complexity to the system than just changes to lncRNA expression.

Of note, the size of H3K9me3 peaks around *slc4a1a* and *fbxo32* were approximately the size of a single nucleosome (150–200 bp), as were many of the peaks in and around protein coding genes. There were also larger heterochromatin domains containing broader peaks and these typically occurred around unannotated transcripts (Additional file 13). In certain instances, the broad peaks had diurnal changes, but there were no apparent oscillations in the corresponding transcript.

## Discussion

Using multiple genomic approaches, the work reported here was designed as an initial test for the hypothesis that diurnal gene expression and age-related dysregulation is driven, in part, by lncRNA-guided heterochromatin changes. This hypothesis arose from a number of previous observations. First, H3K9me2 and H3K9me3-dependent facultative heterochromatin occurs at the clock genes during the repressive phase of the circadian cycle [24, 40, 42]. Second, the Per-complex contains KMT1/SUV39 along with a number of noncoding RNA binding proteins including NONO [24, 27]. Third, the *Per2* gene has an antisense transcript and in Neurospora, the *frq* NAT natural antisense transcript (NAT) *qrf* is needed for H3K9me-dependent facultative heterochromatin [31, 32, 39]. Forth, there are multiple examples, from single-cell eukaryotes to mammals that reveal H3K9me3 can be directed by lncRNA ranging from centromeres in *S. pombe* [59] to H19-guided imprinting in mammals [60]. And finally, emerging models on aging suggest there is a redistribution of H3K9me3 and DNA methylation, which is either a direct cause or a consequence of aging. Taken together, these findings suggest age-related changes to the circadian transcriptome may occur in part due to age-related changes to circadian lncRNA, which could then alter the underlying heterochromatin. The data presented here is by no means definitive proof this as an all-inclusive model, but does demonstrate a likelihood this occurs at some loci. The data also reveal other mechanisms must exist to account for changes in gene expression independent of H3K9me3.

In good agreement with previous observations, this report shows global reprogramming of the circadian transcriptome during aging even though the underlying circadian oscillator remains largely unaffected. For example, in the liver, age-induced changes in circadian expression are accompanied by changes in protein acetylation and NAD+ metabolism [14]. Similarly, extensive reprogramming of circadian transcriptome in aged stem cells causes a switch in expression of genes involved in homeostasis, to those involved in tissue-specific stress such as DNA damage or inefficient autophagy [13]. Our study contributes new insights into mechanisms underlying age-related changes to diurnal expression and further implicates epigenetic changes that include alterations to circadian lncRNA and genome-wide heterochromatin as potential regulators.

An additional objective of this study was to further identify lncRNAs in zebrafish brain. Considering the majority of expressed transcripts in mammals do not appear to code for proteins [61], these data should serve as a valuable resource to the zebrafish community. In particular, lncRNAs have diverse roles in regulation ranging from transcriptional interference in yeast, to X chromosome inactivation, imprinting, and epigenetic silencing in mammals, including directing H3K9me3 and H3K27me3 [62] so a more complete zebrafish noncode is warranted. Due to the growing evidence lncRNAs are targets of circadian regulation in a variety of tissues and organisms, it is important to get a base-line measure of lncRNAs to begin to understand their role in the clockworks. For example there are 112 lncRNAs showing differential expression in the light and dark in rat pineal gland [63] and mouse circadian transcriptome data suggests there are at least 1000 circadian regulated lncRNAs observed in multiple tissues [30]. Here, we expanded the zebrafish noncode by adding brain to a developmental dataset and explored whether any of these lncRNAs coincided with diurnal changes in H3K9me3. Further, we examined how lncRNA expression changed with age and time-of-day. Among the over 140,000 transcript isoforms identified via Cufflinks, more than 50% were previously unannotated in the GRCz10 and 50,524 are predicted to be lncRNAs by PLEK (both long intergenic non-coding RNA and OT). It remains to be determined how many of these newly identified transcripts are under direct control of the circadian clock and whether they function as heterochromatin regulators in either *cis* or *trans*.

Our findings of age-related changes to diurnal transcription are consistent with other reports showing the core circadian oscillator is relatively stable regardless of age, but *ccg* expression undergoes chronological changes over an animals lifetime. In this study, not only are protein-coding *ccgs* changing with age, but also a significant number of rhythmic lncRNA also change as organism’s age. From gene ontology analysis of protein-coding genes, it is clear the circadian transcriptome changes from anabolic to catabolic processes as one ages. The dynamic nature of age-related changes to clock output between 4 M and 12 M suggests the existence of a complex regulatory mechanism that partially resides at the level of chromatin and becomes unstable with age (20 M). This is quite consistent with the age-related changes in acetylation given deacetylation precedes heterochromatin.

We also examined age-related changes in diurnal genes by disease ontology analysis in hopes of identifying key factors that could provide insight into age-related disease. We specifically examined changes in the expression of Werner syndrome helicase (WRN) because mutations in WRN protein cause an advanced aging disorder known as adult progeria. WRN, like PER2, is associated with KMT1 (SUV39h) and HP1 and there is a reduction in heterochromatin in WRN null cells [47]. We did not observe any age-related changes to WRN (data not shown), nor did it have a diurnal expression (at least at the phase we examined). However, WRN interacting protein 1 (*wrnip1*) was lost with age (Additional file 8c), so it is interesting to speculate that an interaction between Wrn and KMT1 might occur through Wrnip1 and this could potentially account for some of the age-related changes in heterochromatin. Alternatively, other alterations in heterochromatin may be mediated by changes in lncRNAs that potentially interact with the Per2 complex and direct Per-associated KMT1 to different loci; a speculative notion at this stage, but one that needs further study. Regardless, it is clear there is a complex regulatory system below the level of the master oscillator controlling circadian output that changes with age and occurs concurrent with changes in lncRNA, H3K9me3, and must include other chromatin changes.

As a final note, it is important to emphasize that the goal of this study was not to identify circadian protein-coding genes per se, this has been done exhaustively by others. As such, we intentionally ignored established guidelines for circadian rhythm research [64] and instead set out to examine a potential mechanism for age-related changes to gene expression, with the main focus being lncRNA discovery and heterochromatin. To that end, we limited our analysis to two diurnal time points, sequenced to extremely high depth (90 million paired-end reads), and preserved strandedness to augment transcript discovery. This focus on diurnal regulation proved fruitful as it allowed us to identify a much broader range of lncRNAs and analyze H3K9me3 in greater detail. It is now clear that a more comprehensive analysis of age-related changes to lncRNAs and corresponding chromatin structural changes on the circadian time scale is warranted to uncover more detailed mechanism(s) underlying aging.

## Conclusions

There are age-related changes to lncRNA and H3K9me3 that coincide with changes in *ccg* expression. Based on the known function of lncRNA guiding epigenetic modifications in cellular events such as imprinting, X-chromosome inactivation, and NAT-mediated heterochromatin, it is possible that a subset of diurnal lncRNA may guide age-related epigenetic changes that affect *ccg* expression without altering the master circadian oscillator. This theory is supported by our observations showing diurnal and age-related changes in gene expression, including lncRNAs, coincide with diurnal and age-related changes in H3K9me3. This notion provides one potential solution to the perplexing observation that clock output is dynamic as organism’s age, but the core clock does not change appreciably [13, 14]. Our findings reveal that chromatin structure, in the form of circadian-regulated facultative heterochromatin, is one facet that determines whether a gene is rhythmic in young or older adults or when it is expressed in given block of time over one’s lifespan. Based on these and other findings, this may be one of many underlying causes of age-related disease manifestation observed with circadian disruption.

## Methods

### Animal care

Wild-type zebrafish (*Danio rerio)* (SAT) were obtained from ZIRC (Zebrafish International Resource Center, Oregon), housed in system water (conductivity 600 ppm, pH 7.4) at 27 C, and fed twice daily. For diurnal entrainment, the fish were placed under a 12:12 light:dark cycle, but otherwise maintained at 14:10 light:dark cycle for breeding. Embryos and young larvae were raised in egg water (30 mg/L instant ocean in deionized water). For tissue extraction, fish were sacrificed by emersion in cold MS-222 (300 mg/l, Sigma), decapitated and dissected under Phosphate Buffered Saline (PBS) using microdissection tools.

### RNA-Seq

Four Month (M), 12 M and 20 M zebrafish were maintained under a 12:12 light:dark cycle for one week prior to tissue removal. Zebrafish were sacrificed at ZT4 and ZT16 and brain tissue was removed as described [65]. Tissue was immediately snap frozen in liquid nitrogen and RNA was extracted from all samples simultaneously using TRIzol (Invitrogen). RNA-Seq libraries (*n* = 2) were processed at the Columbia Genome Center (New York, NY). Briefly, ribosomal RNA was depleted with RiboZero Gold (Illumina) and converted to cDNA with TruSeq Stranded Library Kit (Illumina). The libraries were sequenced on an Illumina 2500 to a depth of 90 million 100 bp paired-end reads per library. The data are deposited in Gene Expression Omnibus (GEO) under the accession number GSE109856.

### ChIP-Seq

Zebrafish were maintained under identical conditions described for the RNA-seq. Brain tissue was removed and immediately cross-linked with 1% formaldehyde for 10 min then quenched with 0.1 M glycine for 10 min prior to being snap frozen in liquid nitrogen. Frozen tissue was lysed by mechanical disruption in the presence of ChIP lysis buffer [50.0 mM Hepes (pH 7.4), 150 mM NaCl, 1.0 mM EDTA, 1% Triton X-100, 0.1% deoxycholate] containing protease inhibitors (2.0 μg/mL leupeptin, 2.0 μg/mL pepstatin A, 1.0 mM PMSF). Additional cell disruption and chromatin shearing were done in a Misonix cup sonicator 5 times for 30 s at 20% power. The resulting lysates were cleared of cellular debris by centrifugation at 16,000 x g for 1 min. The average size of sheared chromatin was 500 bp. For the ChIP, 200 μg of lysate was mixed with 3 μg H3K9me3 antibody (Abcam ab8898) prebound to Protein A magnetic beads (Dynal) then incubated overnight at 4 °C. The ChIP samples were washed 5x with RIPA buffer (10 mM Tris pH 8.0, 1 mM EDTA, 1% Triton X-100, 0.1% SDS, 0.1% sodium deoxycholate, 140 mM NaCl) and eluted with 0.1 M sodium bicarbonate, 1% SDS. Isolated samples were heated at 65 °C to reverse crosslinks and protein was removed by the addition of proteinase K. DNA was further purified by phenol-chloroform extraction then precipitated with 1/10 volume 3 M NaOAc pH 5.4 and 2.5 volumes 95% ethanol. Purified DNA from each H3K9me3 ChIP done in triplicate was used for library preparation. DNA was sequenced 50 million 50-bp single-end reads on the Illumina HiSeq 2500 at Duke University Genome Center. The data are deposited in Gene Expression Omnibus (GEO) under the accession number GSE109856. H3K9me3 from the ChIP-seq was validated by qPCR using oligonucleotides contained in Additional file 14.

### RNA-seq analysis

The RNA-seq data were processed using two different analysis pipelines roughly outlined in Additional file 1: Figure S1 and S2 for transcript discovery. The first method used Tophat2-Cufflinks-Cuffdiff and second used Hisat2-Stringtie-Cuffdiff [48, 49, 66]. To enhance transcript discovery, we also included previously published RNA-seq dataset from developing zebrafish embryos (GSE32989) [50]. The combined analysis yielded a single GTF file for differential expression. Paired-end reads were aligned to zebrafish reference assembly GRCz10 (http://www.ensembl.org/Danio_rerio/Info/Index) with TopHat2 (version 2.0.9) using the GRCz10 GTF file as a guide. The mapped reads were assembled with Cufflinks (version 2.2.1). All transcripts from Cufflinks were combined to create new GTF file using Cuffmerge with the default FPKM cutoff of 0.05. The differential gene expression was performed using Cuffdiff [48]. Data was also mapped with HISAT2, and a separate transcript file was created using StringTie [49]. The StringTie minimum FPKM was set 0.1 because that was the maximum level capable of detecting the known *Per2AS* transcript. All subsequent statistical analyses were done in R Studio using the bioconductor package CummeRbund. Visualization of transcripts was done using the Integrative Genomics Viewer (IGV) [67].

### ChIP-seq analysis

The reads of H3K9me3 ChIP-seq were mapped to the zebrafish GRCz10 genome using Bowtie2 [68]. Bam files of each triplicate were further processed using MACS2 for peak calling (FDR ≤ 0.01) [57]. Differential H3K9me3 enrichment between different conditions was analyzed by Diffbind and visualized with MAplot [58]. ChIPseeker was used to analyze genomic features of the H3K9me3 enrichment [69]. The H3K9me3 peaks were visualized with IGV [67].

### Quantitative reverse transcription PCR

Zebrafish (*n* = 3) were maintained and entrained under identical conditions described for RNA-seq. Brain tissue was removed and immediately snap frozen in liquid nitrogen. TRIzol reagent was used for the extraction of total RNA from the brain tissue. 500 μg of total RNA was then used as a template with the SuperScript III First-Strand Synthesis kit (Invitrogen) to produce cDNA. The specific targets were amplified by RT-PCR using oligonucleotides in Additional file 14 using *hmbs* as the internal control. T-tests were performed to calculated *p*-value to determine significant difference between samples.

### Gene and Disease ontology

WebGestalt (WEB-based GEne SeT AnaLysis Toolkit) was used for Gene Ontology (GO) enrichment using an adjusted *p*-value ≤0.01 as the cutoff, with multiple-hypothesis correction to calculate the enrichment *p*-value. The GO clusters with significant *p*-value were taken for further analysis. The top GO terms were converted to log_10_ adjusted *p*-value and visualized with a radial graph or in tabular format [52, 53]. In order to use human Disease Ontology database, the zebrafish genes were first matched to their human orthologues by using Ensembl biomart (www.ensembl.org/biomart) and we used Bioconductor DOSE packages with *p*-value ≤0.05 cut to identify corresponding genes.

## Additional files

Additional file 1: TopHat2/Cufflinks revealed 146,910 transcripts/isoforms with the min-isoform-fraction (-F) set to 0.05 (default setting). 16,532 out of the 146,910 isoforms were known protein-coding genes (class code =). Overlapping transcripts (OT) were identified using Cuffcompare by extracting transcripts with class code x and s from the merged GTF file. Transcripts with class code i, o and u were also extracted and further processed using Bedtools intersect (option -S) to sort antisense OT. In total, there were 14,774 OT fitting these criteria. The 14,774 OT were subjected to PLEK analysis to predict lncRNAs. PLEK revealed 5299 OT lncRNAs. 75% of the OT lncRNAs are unannotated intergenic transcripts with class code u. The remaining 25% are lncRNAs that overlap with an exon of a protein-coding gene. Of note, after PLEK, there were no single exon transcripts. (EPS 1498 kb)

Additional file 2: The HISAT2/StringTie analysis revealed 318,442 transcripts/isoforms with FPKM cutoff set to 0.1 in StringTie. This cutoff, which was an order a magnitude lower than the default, was necessary to identify the known *per2AS* transcript. HISAT2/StringTie identified 15,245 protein-coding transcripts (class code =). OTs were identified using a similar pipeline as described in Additional file 1: Figure S1 except we used Gffcompare instead of Cuffcompare. Transcripts with class codes x, s, i, o or u were extracted and those with class code i, o, or u, were processed with Bedtools intersect. In all, this revealed 183,635 potential OT. The 183,635 OTs were processed using PLEK yielding 131,635 predicted lncRNAs. However, after removing single exon transcripts, only 31,629 OTs remained. Of these, 76% were intergenic and the remaining 24% were contained within another gene. (EPS 1699 kb)

Additional file 3: Expression patterns of lncRNAs from early development through adult aged zebrafish. To enhance transcript discovery, we included stranded RNA-seq data from zebrafish embryogenesis [50]. In addition to allowing enhanced detection of lncRNAs, this dataset provided the ability to examine dynamic changes in lncRNA expression from bud stage to 20 M old fish. To identify all the lncRNAs, we filtered the RNA-seq data using the flowchart in panel a. We started with the 146,910 transcripts from Cufflinks and found 86,327 undefined transcripts (class code u). Next, PLEK predicted 50,524 potential lncRNA. The 50,524 transcripts were further filtered by their expression changes based on the criteria that a transcript must have a statistical change in expression in at least one-time point. This analysis yielded 17,702 unique lncRNAs. The 17,702 transcripts were then clustered based on similarities in the timing of expression. (b-i) Expression patterns of the 17,702 lncRNA from the bud stage of embryogenesis to 20 M fish segregated into eight groups based on similarity in expression. The thick black lines are the average pattern for each group. Also shown is a representative transcript from each group visualized with IGV (left panels). Certain lncRNAs were largely restricted to adult fish ≥4 M (Groups 1, 3, 5 and 6) while others were largely restricted to developmental stages (Groups 2 and 8). Group 7 appeared to span the later stages of development into adult fish ≤12 M. In contrast, Group 4 lncRNAs were expressed at every time point. Of note, the lncRNAs in Group 6 were largely restricted to adult fish and acquired a diurnal change when the fish were 12 M old. (EPS 6739 kb)

Additional file 4: Diurnally expressed genes at 4 months. (XLSX 24 kb)

Additional file 5: Diurnally expressed genes at 12 months. (XLSX 41 kb)

Additional file 6: Diurnally expressed genes at 20 months. (XLSX 30 kb)

Additional file 7: Age-dependent expression changes in diurnal genes. To gain insight into how diurnal transcripts changed with age, we clustered transcripts based on diurnal expression and compared them across the other age groups. The heatmaps represent log_2_ fold change in FKPM values of diurnal genes between ZT4 and ZT16 and cross-compared them to the same transcripts at the different ages. (a) diurnal transcripts at 4 M compared to 12 M and 20 M. (b) Diurnal transcripts at 12 M compared to 4 M and 20 M (c) Diurnal transcripts at 20 M compared to 4 M and 12 M. The log_2_ fold change comparing ZT4 to ZT16 is plotted as a bar chart on the top of each heatmap. (EPS 1701 kb)

Additional file 8: Disease Ontology enrichment analysis of diurnal transcripts at different ages. We performed DO on protein-coding genes that had diurnal expression. (a) The top-ranked DO terms of diurnal genes in the specific age are illustrated in a dot graph (*p* ≤ 0.05). (b) Bar plots of *complement component 3a* (*c3a.1*) and *annexin* 3b (*anxa3b*). Expression changes in both *c3a.1* and *anxa3b* correlate with age-related cognitive decline [56]. (c) We also examined the expression of *wrnip1* and found that it is lost with age. (EPS 1310 kb)

Additional file 9: Expression of *myl10*, *cishb*, and *myl1* sense and antisense transcripts. IGV illustration showing relative expression of sense and antisense transcripts originated from (a) *myl10*, (b) *cishb* and (c) *myl1* for both time points for all three age-groups. The panel on the right shows relative expression validated by RT-PCR (*n* = 3). Error bars represent the SEM with asterisks indicating *p*-value < 0.05. (EPS 1721 kb)

Additional file 10: Genes that changed expression between 4 M and 20 M at ZT4. (XLSX 27 kb)

Additional file 11: Genes that changed expression between 4 M and 20 M at ZT16. (XLSX 38 kb)

Additional file 12: Expression and H3K9me3 for *slc4a1a*. (a-b) The expression of *slc4a1a* sense and antisense transcripts was confirmed by RT-PCR (*n* = 3). (c) H3K9me3 ChIP-qPCR at *slc4a1a.* Error bars represent the SEM and asterisks indicates *p* < 0.05. (EPS 962 kb)

Additional file 13: Representative view of H3K9me3 broad peaks. Broad peaks over 1 kb around unannotated transcripts were observed and illustrated in the IGV viewer. The H3K9me3 broad peaks were found in an unannotated transcript XLOC_030565 (a) and downstream of an unannotated transcript XLOC_020820 (b). The broader peaks spanning multiple nucleosomes showed a diurnal rhythm between ZT4 and ZT16. (EPS 932 kb)

Additional file 14: Oligonucleotides used in this report. (EPS 1201 kb)

## Abbreviations

ChIP-seq: Chromatin Immunoprecipitation with DNA sequencing
CRFH: Circadian regulated facultative heterochromatin
DO: Disease Ontology
GO: Gene ontology
H/S: HISAT2/StringTie, PLEK
lncRNA: Long noncoding RNA
OT: Overlapping transcripts
RNA-seq: RNA sequencing
T/C: Tophat2/Cufflinks
